# Evaluation of print angle on accuracy of 3D printed dental models

**DOI:** 10.3389/fdmed.2026.1931793

**Published:** 2026-08-26

**Authors:** Aarthi Chand, Hashmi K. M, K. Saidath

**Affiliations:** Nitte (Deemed to be University), AB Shetty Memorial Institute of Dental Sciences (ABSMIDS), Department of Orthodontics and Dentofacial Orthopaedics, Mangalore, India

**Keywords:** 3D printing, build orientation, dental models, dimensional accuracy, print angle

## Abstract

**Introduction:**

Additive manufacturing, introduced by Charles Hull through stereolithography, enables the fabrication of three-dimensional (3D) structures with high precision by curing material layer by layer. In dentistry, print orientation significantly influences the dimensional accuracy and mechanical properties of 3D-printed models used for surgical splints, aligners, drill guides, and other orthodontic applications. This study evaluated the effect of different print orientations on the dimensional accuracy of maxillary and mandibular dental models fabricated using a Digital Light Processing (DLP) printer.

**Aim and objective:**

To evaluate the effect of different 3D printing angles on the dimensional accuracy of dental models fabricated using a Digital Light Processing (DLP) printer.

**Materials and methods:**

Type IV gypsum dental casts were scanned using the 3Shape OrthoAnalyzer and printed at four orientations (0°, 30°, 60°, and 90°) using a DLP printer. The printed models were washed, post-cured, rescanned, and digitally superimposed onto the original scans to assess dimensional accuracy. Statistical analysis was performed using ANOVA followed by Tukey's *post hoc* test, with a significance level of *p* < 0.05.

**Results:**

Print orientation significantly affected the dimensional accuracy of maxillary models, with significant differences observed among selected orientation groups. No significant differences were found among mandibular models printed at different orientations. Significant differences were also observed between maxillary and mandibular models printed at 30° and 60°.

**Conclusion:**

Maxillary models printed at 30° and 60° demonstrated greater dimensional accuracy than those printed at 0° and 90°. Although print orientation influenced accuracy, all deviations were within clinically acceptable limits, indicating that orientation may be selected based on clinical requirements, printing time, resin usage, and build platform efficiency.

## 1 Introduction

Additive manufacturing has revolutionized dentistry by enabling the fabrication of highly accurate three-dimensional (3D) structures through a layer-by-layer manufacturing process. Since the introduction of stereolithography by Charles Hull, 3D printing has evolved from its initial applications in engineering and aerospace to become an integral component of modern dental practice. Today, additive manufacturing is extensively employed in orthodontics, prosthodontics, implantology, and maxillofacial surgery for the fabrication of diagnostic models, surgical guides, occlusal splints, aligners, temporary restorations, and customized appliances (1).

Among the various 3D printing technologies available, Digital Light Processing (DLP) has gained considerable popularity because it polymerizes an entire resin layer simultaneously, thereby reducing printing time while maintaining high precision. The ability of DLP printers to produce detailed models with minimal manufacturing time has made them particularly suitable for orthodontic applications, where dimensional accuracy is essential for successful treatment outcomes.

The accuracy of a 3D-printed dental model is influenced by several printing parameters, including layer thickness, exposure time, resin characteristics, build orientation, support structures, post-processing procedures, and printer calibration. Among these variables, print orientation is considered one of the most important determinants of dimensional accuracy, mechanical strength, surface finish, resin consumption, and printing efficiency. Variations in build orientation alter the direction of layer formation and the distribution of polymerization stresses, thereby influencing the fidelity of the printed model.

Accurate dental models are indispensable for fabricating clear aligners, surgical splints, indirect bonding trays, retainers, and implant guides (2). Even minor dimensional discrepancies may result in improper appliance fit, compromised force delivery, inaccurate tooth movement, or reduced clinical efficiency. Consequently, optimization of printing parameters is necessary to ensure predictable and reproducible treatment outcomes (3).

Previous studies have demonstrated conflicting results regarding the ideal print orientation (4–7). While some investigations have reported superior accuracy at lower build angles, others have favoured intermediate or higher angulations depending on the printing technology, resin, and clinical application. Furthermore, most available studies have evaluated only maxillary models or stereolithography (SLA) printers, leaving limited evidence regarding the influence of print orientation on both maxillary and mandibular dental models fabricated using Digital Light Processing (DLP) technology (8–23).

The anatomical differences between the maxillary and mandibular arches may influence the behaviour of printed models during fabrication and post-processing. Therefore, it cannot be assumed that the optimal build orientation for one arch will necessarily produce similar accuracy in the other. Understanding these differences is important for selecting the most appropriate print orientation for routine clinical practice.

Considering the increasing adoption of digital workflows in orthodontics and the limited evidence available regarding DLP-printed dental models, the present study was undertaken to evaluate the effect of different print orientations (0°, 30°, 60°, and 90°) on the dimensional accuracy of maxillary and mandibular dental models fabricated using a Digital Light Processing printer. The findings of this study may assist clinicians in selecting an appropriate build orientation that optimizes printing accuracy while maintaining clinical efficiency.

## 2 Aim and objectives

### 2.1 Aim

To evaluate the effect of different three-dimensional (3D) printing angles on the dimensional accuracy of maxillary and mandibular dental models fabricated using a Digital Light Processing (DLP) printer.

### 2.2 Objectives

- To fabricate maxillary and mandibular dental models at four different print orientations (0°, 30°, 60°, and 90°) using a DLP printer.
- To assess the dimensional accuracy of the printed models by comparing them with the original Standard Tessellation Language (STL) files through digital superimposition.
- To compare the dimensional deviations among the four print orientations for both maxillary and mandibular models.
- To determine the print orientation that produces the highest dimensional accuracy.
- To compare the influence of print orientation on the dimensional accuracy of maxillary and mandibular dental models.

## 3 Materials and methods

### 3.1 Study design

The present *in vitro* study was conducted in the Department of Orthodontics and Dentofacial Orthopaedics, A. B. Shetty Memorial Institute of Dental Sciences, after obtaining approval from the Institutional Ethics Committee (ETHICS/ABSMIDS/203/2022). The study evaluated the influence of different print orientations on the dimensional accuracy of maxillary and mandibular dental models fabricated using a Digital Light Processing (DLP) printer.

### 3.2 Sample size

The sample size was calculated using nMaster version 2.0 software based on previous literature. A total of 112 printed dental models were included in the study, with 14 samples in each subgroup, ensuring adequate statistical power for comparison among the different print orientations.

### 3.3 Materials

**Figure 1:**
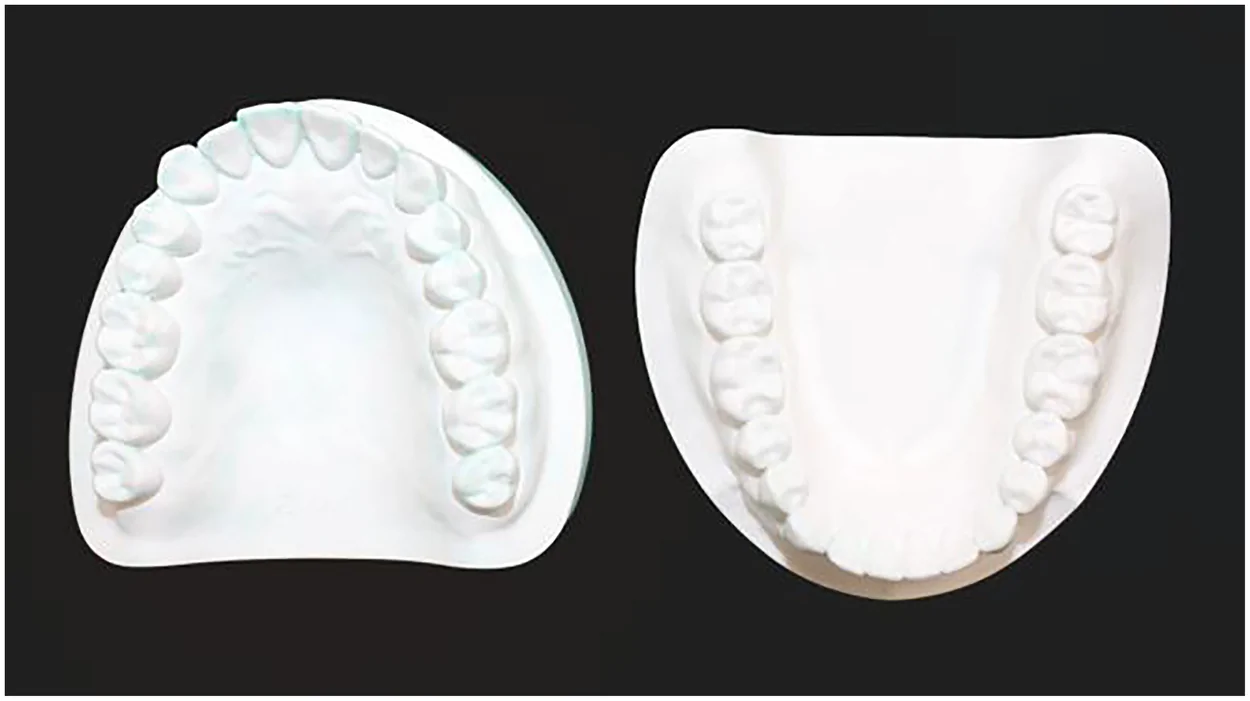
Study casts.

**Figure 2:**
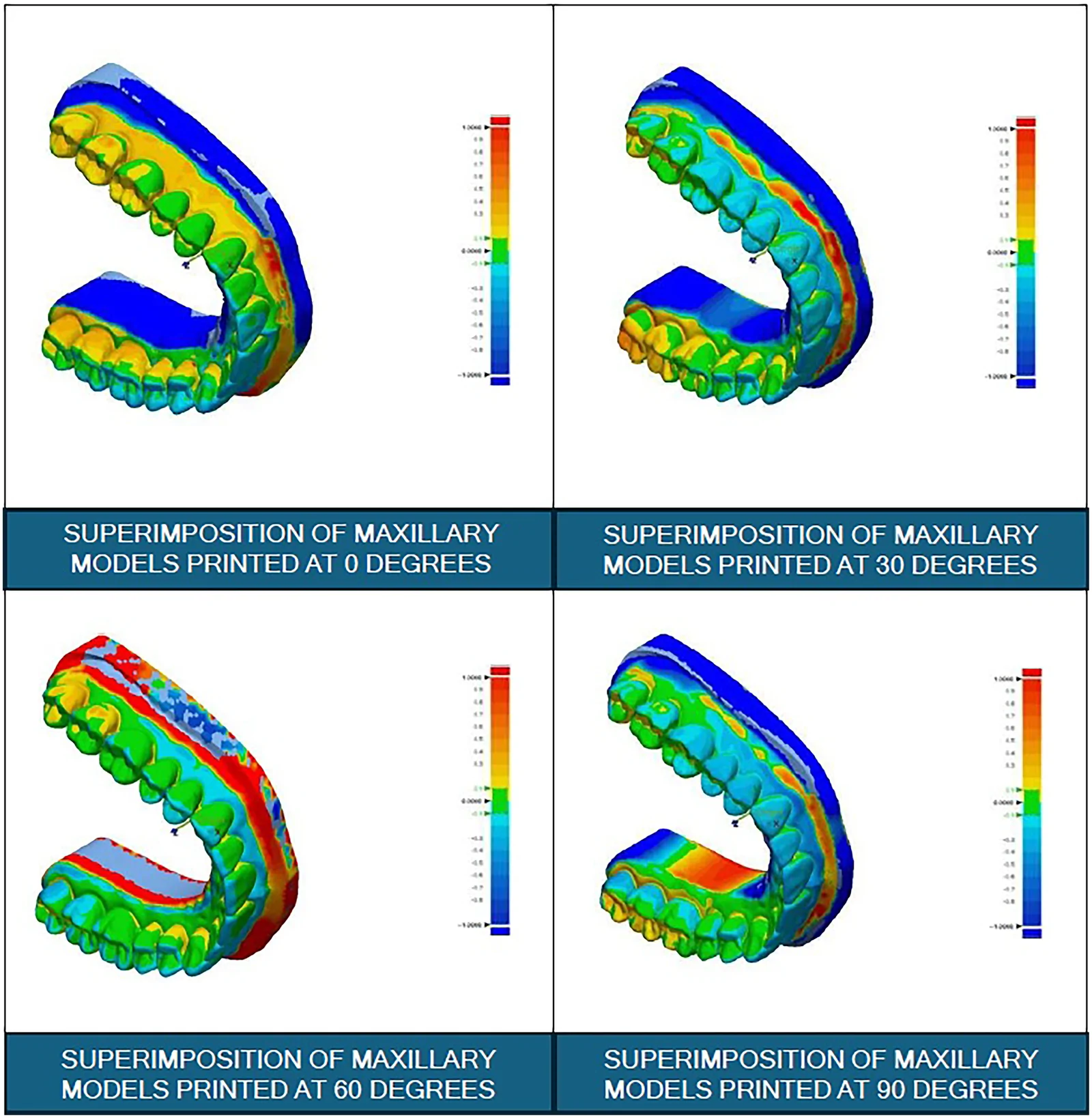
Superimposition of maxillary models.

**Figure 3:**
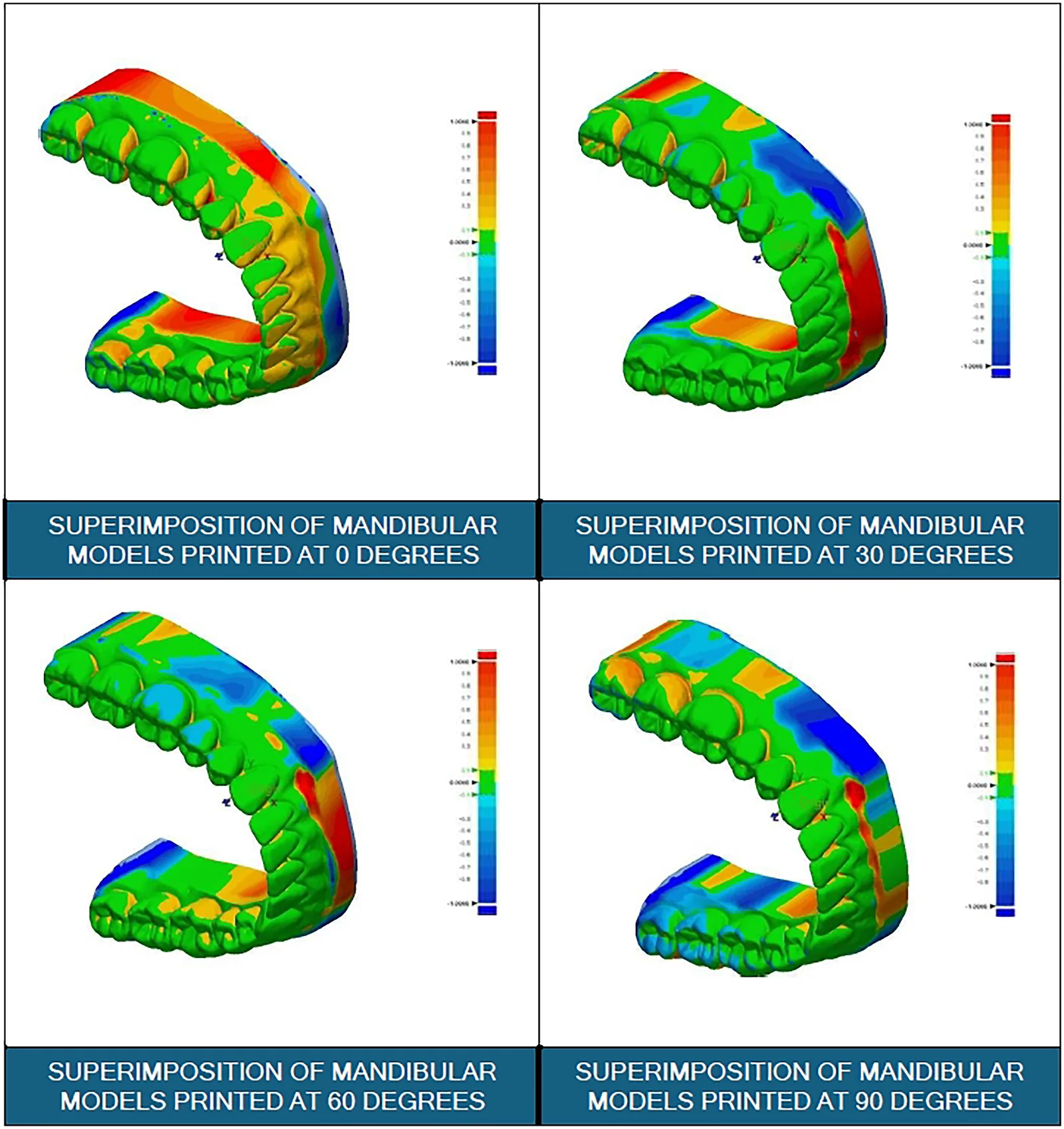
Superimposition of mandibular models.

The materials and equipment used in the study included:

- Type IV dental stone for fabrication of reference casts (Figure 1)
- Ideal maxillary and mandibular cast moulds
- 3Shape TRIOS intraoral scanner for digital scanning
- 3Shape OrthoAnalyzer software for model orientation
- SprintRay Pro Digital Light Processing (DLP) 3D printer
- SprintRay ProWash/Dry unit for post-print cleaning
- SprintRay ProCure unit for post-polymerization curing
- GeoMagic Control × software for three-dimensional superimposition and deviation analysis (Figures 2, 3)

### 3.4 Methodology

Reference maxillary and mandibular dental casts were fabricated using Type IV gypsum and allowed to set under standardized laboratory conditions (Figure 1). To standardise the kind of model that would be printed, we have used a dental model of a Class I occlusion with a 2 mm overjet and a 2 mm overbite, exhibiting complete alignment without spacing. Each cast was scanned using a 3Shape TRIOS intraoral scanner to generate a master Standard Tessellation Language (STL) file representing the reference model.

The STL files were imported into 3Shape OrthoAnalyzer software, where a standardized reference plane was established using anatomical landmarks. The digital models were trimmed uniformly to ensure consistency before printing.

The master STL files were printed using a SprintRay Pro DLP printer at four different build orientations: 0°, 30°, 60°, and 90°. Fourteen maxillary and fourteen mandibular models were fabricated for each print orientation, resulting in eight experimental subgroups.

The printer was calibrated according to the manufacturer's recommendations before each printing cycle. Automatic support structures were generated for the 30° and 60° print orientations, whereas the 0° and 90° models were printed directly on the build platform. Printing order was randomized to eliminate systematic bias. The 0° and 90° orientation did not have support beams. However, this will not affect the study as we have not included the base model in the assessment. The reason that supported and an unsupported model would have different accuracy is because the adhering layer requires additional exposure time to ensure its safe adhesion to the platform and causes compression, which consequently leads to a shortened total height. Since we have used points from the buccal surfaces of the teeth rather than the base, the superimposition would not be affected by the support.

Following fabrication, all printed models underwent standardized post-processing. Excess resin was removed using the SprintRay ProWash/Dry unit, followed by final polymerization in the SprintRay ProCure unit according to the manufacturer's protocol.

Each printed model was rescanned using the same intraoral scanner to generate STL files under identical scanning conditions. These files were processed using the same orientation protocol as the reference models to eliminate procedural variation.

Dimensional accuracy was assessed by digitally superimposing each printed STL file onto the corresponding master STL file using GeoMagic Control ×  software. Colour deviation maps were generated to evaluate the agreement between the printed and reference models. Positive and negative deviations beyond ±0.25 mm were considered outside the clinically acceptable range. The GeoMagic software does not measure mean deviation, it measures the deviation from the original model. The way that it does, this is by super imposing both of the scan images and detecting the amount of deviation between both of the scan images. The alignment method was done using the software, by super imposing both of the scans. The points used for deviation in the study were the gingival third of the buccal surface from 3rd molar to 3rd molar on either side. Hence, 16 points on the model were selected to assess the deviation. The model base was not included (Figures 2, 3).

### 3.5 Outcome measure

The primary outcome measure was the dimensional deviation between the printed dental model and the original reference STL file following three-dimensional superimposition.

### 3.6 Statistical analysis

All data were entered into Microsoft Excel and analysed using SPSS version 23. Descriptive statistics were expressed as mean and standard deviation. Intergroup comparisons of dimensional deviation were performed using one-way Analysis of Variance (ANOVA), followed by Tukey's *post hoc* test for multiple comparisons. Differences between maxillary and mandibular models at corresponding print orientations were analysed using the independent t-test. A *p*-value <0.05 was considered statistically significant.

## 4 Results

A total of 112 dental models (56 maxillary and 56 mandibular) were fabricated using a Digital Light Processing (DLP) printer at four build orientations (0°, 30°, 60°, and 90°). The printed models were rescanned and digitally superimposed with the corresponding master Standard Tessellation Language (STL) files to evaluate dimensional accuracy. The deviations obtained for all groups were within the predetermined clinically acceptable limit of ±0.25 mm, indicating that every print orientation produced models suitable for clinical use.

### 4.1 Comparison of dimensional accuracy of maxillary dental models

The mean deviation of the maxillary models was 0.1215 mm at 0°, 0.0862 mm at 30°, 0.0967 mm at 60°, and 0.1234 mm at 90°. Among the four orientations, 30° exhibited the least deviation, followed by 60°, indicating superior dimensional accuracy, whereas 90° showed the greatest deviation. The comparison among the four groups revealed a statistically significant difference. *Post hoc* analysis demonstrated significant differences between the 0° and 30°, 0° and 60°, 30° and 90°, and 60° and 90° groups, while no significant difference was observed between the 0° and 90° or 30° and 60° groups. These findings suggest that intermediate print orientations produced more accurate maxillary models than extreme orientations (Supplementary Data Sheet 1).

### 4.2 Comparison of dimensional accuracy of mandibular dental models

The mean deviations recorded for mandibular models were 0.1251 mm at 0°, 0.1098 mm at 30°, 0.1188 mm at 60°, and 0.1217 mm at 90°. Although the 30° orientation demonstrated the lowest mean deviation, one-way ANOVA showed no statistically significant difference among the four print orientations. Therefore, print angle did not significantly influence the dimensional accuracy of mandibular dental models (Supplementary Data Sheet 1).

### 4.3 Comparison between maxillary and mandibular dental models

Comparison of the two arches at corresponding print orientations demonstrated no statistically significant difference between maxillary and mandibular models printed at 0° and 90°. However, significant differences were observed at 30° and 60°, where the maxillary models showed lower mean deviations than the mandibular models, indicating greater dimensional accuracy. The greatest inter-arch difference was observed at the 30° print orientation, followed by the 60° orientation (Supplementary Data Sheet 1).

#### 4.3.1 Summary of results

The present study demonstrated that print orientation significantly influenced the dimensional accuracy of maxillary dental models but not mandibular models. Among all orientations evaluated, 30° provided the highest dimensional accuracy, followed by 60°, whereas 0° and 90° exhibited comparatively greater deviations. Despite these differences, all printed models remained within clinically acceptable limits, indicating that each print orientation is suitable for clinical application, with intermediate orientations offering improved accuracy (Supplementary Data Sheet 1).

## 5 Discussion

Three-dimensional printing has become an integral component of digital dentistry and is increasingly used across multiple dental specialties, including orthodontics. The accuracy of printed dental models directly influences the fit of orthodontic appliances, clear aligners, surgical splints, and implant guides. Among the numerous factors affecting printing accuracy, build orientation has received considerable attention because it influences layer formation, polymerization shrinkage, support distribution, printing time, and resin consumption. The present study evaluated the effect of four print orientations (0°, 30°, 60°, and 90°) on the dimensional accuracy of maxillary and mandibular dental models fabricated using a Digital Light Processing (DLP) printer.

Digital Light Processing (DLP) technology was selected for evaluation in the present study because it is one of the most widely used 3D printing techniques in orthodontics, offering an optimal balance between printing speed and dimensional accuracy. Compared with other additive manufacturing technologies, DLP polymerizes an entire resin layer simultaneously, enabling faster fabrication while maintaining high precision. These characteristics make DLP particularly suitable for orthodontic applications, where accurate reproduction of dental anatomy is essential for diagnosis, appliance fabrication, and treatment planning.

Digital superimposition of the printed models with the corresponding master STL files demonstrated that all printed models exhibited deviations well within the clinically acceptable limit of ±0.25 mm. These findings indicate that DLP technology is capable of producing clinically acceptable orthodontic models irrespective of the selected build orientation. Nevertheless, the magnitude of dimensional deviation varied among the different print angles, suggesting that build orientation remains an important determinant of printing accuracy.

In the present study, 30° and 60° print orientations produced significantly greater dimensional accuracy for maxillary dental models than 0° and 90°. Among these, the 30° orientation demonstrated the least mean deviation, indicating the highest accuracy. This observation may be attributed to a more favourable distribution of polymerization stresses and gradual layer formation at intermediate build angles. Angulated printing also permits more uniform support distribution, thereby reducing distortion during fabrication and post-processing.

Conversely, the 0° and 90° orientations exhibited comparatively greater deviations. Models printed parallel to the build platform may undergo compression of the initial layers because of the absence of support structures, whereas vertically oriented models are more susceptible to cumulative layer distortion along the printing axis. Although these orientations demonstrated slightly lower accuracy than intermediate angles, the observed deviations remained clinically acceptable, indicating that they may still be selected when shorter printing time, reduced resin usage, or optimal build-platform utilization is required.

Unlike the maxillary models, mandibular dental models did not demonstrate statistically significant differences among the four build orientations. Although the 30° orientation recorded the lowest mean deviation, the differences were not significant. The comparatively uniform performance of mandibular models may be related to their anatomical morphology, which distributes printing stresses differently during fabrication. These findings suggest that mandibular models are less sensitive to changes in print orientation than maxillary models.

Comparison between the two arches demonstrated significant differences at 30° and 60°, with maxillary models exhibiting greater dimensional accuracy than mandibular models. The anatomical differences between the arches, particularly the broader palatal vault of the maxilla and the comparatively narrower U-shaped mandibular arch, may influence the orientation of printed layers and support distribution, thereby affecting dimensional stability. Consequently, the optimal build orientation for one arch cannot be directly extrapolated to the other.

The present findings are consistent with previous investigations that reported improved dimensional accuracy at intermediate print orientations, particularly 30° and 60°, because these angles provide a favourable balance between layer formation and support configuration. The results also support studies demonstrating that all commonly used print orientations produce deviations within clinically acceptable limits, allowing clinicians to select build angles according to clinical priorities, including printing duration, resin consumption, support removal, and available build-platform space.

Despite the favourable findings, the present study has certain limitations. Only one DLP printer and one printing resin were evaluated; therefore, the results may not be directly applicable to other printing systems or materials. In addition, several factors known to influence dimensional accuracy, including different layer thicknesses, support configurations, resin formulations, and post-curing protocols, were not investigated. Future studies incorporating multiple printers, printing technologies, and larger sample sizes are recommended to validate these findings and establish standardized printing protocols particularly for orthodontic applications where dimensional precision is especially critical.

Overall, the present investigation demonstrated that print orientation influences the dimensional accuracy of DLP-printed dental models, with 30° and 60° orientations producing superior accuracy for maxillary models, while mandibular models showed no significant variation among the tested orientations. Since all deviations remained within clinically acceptable limits, clinicians may select the build orientation based on the specific clinical application while balancing printing accuracy, manufacturing efficiency, resin consumption, and workflow requirements.

The greater dimensional accuracy observed in maxillary models may be attributed to their anatomical configuration. The presence of a broad palatal vault provides increased structural rigidity, allowing polymerization stresses to be distributed more evenly and minimizing deformation during printing and post-processing. Conversely, mandibular models lack this palatal support and possess a thinner U-shaped geometry, making them more susceptible to warpage, shrinkage, and localized distortion.

## 6 Conclusion and future scope

### 6.1 Conclusion

The present *in vitro* study evaluated the effect of four different print orientations (0°, 30°, 60°, and 90°) on the dimensional accuracy of maxillary and mandibular dental models fabricated using a Digital Light Processing (DLP) printer. Dimensional accuracy was assessed by comparing the printed models with the corresponding master Standard Tessellation Language (STL) files using three-dimensional digital superimposition.

Within the limitations of this study, the following conclusions were drawn:

- Print orientation significantly influenced the dimensional accuracy of maxillary dental models, with 30° and 60° build orientations demonstrating greater accuracy than 0° and 90° orientations.
- Among the evaluated print orientations, 30° exhibited the least dimensional deviation, indicating the highest accuracy for maxillary dental models.
- Mandibular dental models did not demonstrate statistically significant differences in dimensional accuracy among the four build orientations, suggesting that print angle had a comparatively smaller influence on mandibular model accuracy.
- A significant difference was observed between maxillary and mandibular models printed at 30° and 60°, indicating superior dimensional accuracy of maxillary models at these intermediate build orientations.
- Although differences in dimensional accuracy were observed among the print orientations, all printed models demonstrated deviations within clinically acceptable limits, confirming that DLP printing can reliably produce accurate dental models for orthodontic applications.

Therefore, selection of print orientation should not be based solely on dimensional accuracy but should also consider printing time, resin consumption, support requirements, build-platform utilization, and the intended clinical application.

### 6.2 Clinical implications

The findings of the present study suggest that 30° and 60° build orientations may be preferred for fabricating highly accurate maxillary orthodontic models, particularly for applications such as clear aligner fabrication, indirect bonding trays, surgical splints, and implant guides. Since all evaluated orientations produced clinically acceptable models, clinicians may select the most appropriate build orientation by balancing dimensional accuracy with printing efficiency, resin consumption, and workflow requirements.

### 6.3 Future scope

Further investigations should include different 3D-printing technologies, including stereolithography (SLA), LCD, and PolyJet systems, to compare dimensional accuracy across various platforms. Future studies should also evaluate the influence of different resins, layer thicknesses, support designs, post-curing protocols, and larger sample sizes. Print speed, base printing, and resin curing affects the accuracy as well as storage period of resin. Temperature and pressure effects, as well as curing methods, curing time and even the maintenance of the resin tank itself can also influence the accuracy of the printing. In addition, assessment of long-term dimensional stability and the accuracy of printed models under routine clinical conditions would contribute to establishing standardized printing protocols for orthodontic practice.

## Data Availability

The original contributions presented in the study are included in the article/Supplementary Material, further inquiries can be directed to the corresponding author.
