# Supplementary material for: Evaluation of print angle on accuracy of 3D printed dental models
Source: Front Dent Med. 2026 Aug 26;7:1931793. doi: 10.3389/fdmed.2026.1931793 (PMC13553454; doi:10.3389/fdmed.2026.1931793)
Supplement: Supplementary file 1 [file Datasheet1.pdf]

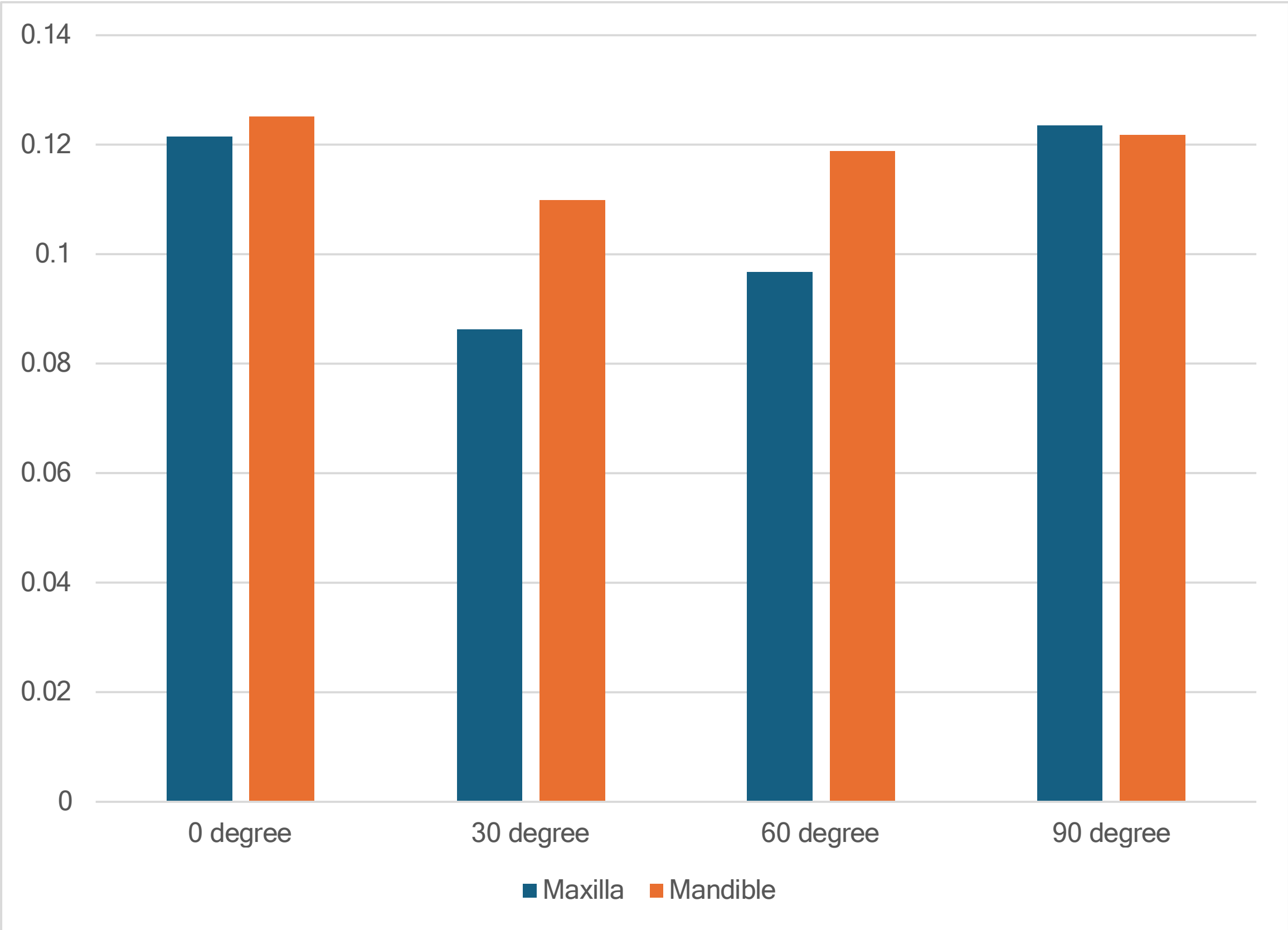

Graph 1. Comparison Of Degree of Deviation Between Maxillary and Mandibular Dental Models Printed In 0^0, 30^0, 60^0 And 90^0 Angulations
